# The Relationship between Knowledge, Dietary Supplementation, and Sleep Quality in Young Adults after the COVID-19 Pandemic

**DOI:** 10.3390/nu15153354

**Published:** 2023-07-28

**Authors:** Maciej Pokarowski, Michal Kedra, Justyna Piwinska, Katarzyna Kurek, Karolina Szczygiel, Piotr Denysiuk, Joanna Popiolek-Kalisz

**Affiliations:** 1 Clinical Dietetics Unit, Department of Bioanalytics, Medical University of Lublin, 20-093 Lublin, Poland; 2 Institute of Psychology, The John Paul II Catholic University of Lublin, 20-950 Lublin, Poland; 3 Experimental Psychopathology Lab, Institute of Psychology, Polish Academy of Sciences, 00-378 Warsaw, Poland; 4 Student Scientific Club at Clinical Dietetics Unit, Medical University of Lublin, 20-093 Lublin, Poland; 5 Department of Cardiology, Cardinal Wyszynski Hospital in Lublin, 20-718 Lublin, Poland

**Keywords:** sleep quality, dietary supplements, sleep knowledge, sleep hygiene

## Abstract

Introduction: Sleep is one of the most important factors affecting the quality of life. More than 50% of Polish adults have sleeping disorders, and young adults are the ones particularly vulnerable to this. This is why the analysis of the predictors of sleep quality, such as sleep hygiene knowledge and dietary knowledge, in young adults is a very important topic, especially after the COVID-19 pandemic, which may have affected sleeping habits. Material and Methods: 402 young adults (mean age 28.12 ± 6.08 years old) were enrolled in the study during the COVID-19 pandemic. Sleep quality was assessed with the Pittsburgh Sleep Quality Questionnaire, while dietary knowledge and sleep hygiene knowledge were assessed with dedicated questionnaires. The participants were also asked about the use of specific dietary supplements. Results: The analysis showed that sleep hygiene knowledge was significantly associated with sleep length (R = −0.17, *p* = 0.003) and overall sleep quality (R = −0.17, *p* = 0.005), while dietary knowledge significantly correlated with time to fall asleep (R = −0.12, *p* = 0.026). The regression analysis revealed that sleep hygiene knowledge was a significant negative predictor of sleep quality impairment (β = −0.13, *p* = 0.028). Significant results were also obtained for the use of supplements (β = −0.20; *p* = 0.001) and the use of medications (β = −0.32, *p* = 0.001), which were negative predictors of sleep quality impairment. Conclusions: This study showed that increased sleep hygiene knowledge promoted improved sleep length and overall sleep quality. In addition, dietary knowledge significantly correlated with time to fall asleep. On the other hand, decreased sleep quality was observed in participants who used medications or dietary supplements.

## 1. Introduction

Sleep is one of the most important factors affecting physical and mental performance and, as a result, affects the quality of life [1]. Sleep allows the autonomic and endocrine systems to regenerate, the blood pressure and heart rate to drop, the overall stimulation of the nervous system to decrease, and the gastrointestinal tract to drop, allowing effective recovery for the body [2]. Epidemiological studies indicate that 50.5% of the Polish population has problems with sleep quality, and sleep disorders are the third most common complaint reported to general practitioners [3]. In addition, the main group vulnerable to sleep deprivation is young adults, especially those associated with college and medical professions [4,5,6]. A key factor affecting the quality of sleep is its duration. It should be adequate for age; for adults, the recommended sleep duration is 7–9 h [7]. Short sleep duration and too much sleep are associated with negative health effects, including total mortality [8,9], cardiovascular diseases [10], diabetes type 2 [11], hypertension [12], respiratory diseases [13], obesity [14,15], and self-rated health [16]. Due to sleep having such a crucial role, it is very important to have proper sleep hygiene, which includes, e.g., management of humidity and temperature in the room where one sleeps, exposure to blue light before bedtime, daytime naps, noise levels during sleep, physical activity directly before the night rest, and alcohol consumption or caffeine intake before bedtime [7,17].

Diet is also an essential aspect of lifestyle. It turns out that a properly balanced and varied diet rich in fruits and vegetables has a significant positive impact on sleep quality [18]. The crucial role of daily carbohydrate supply, especially at dinner, has also been observed, indicating the beneficial effect of carbohydrates with a high glycemic index on sleep latency [19]. In addition, attention should be also paid to products rich in tryptophan in everyday diet [20], such as tart cherries, kiwi, and warm milk [21,22,23,24].

Sleep problems may contribute to individuals searching for dietary supplementation that improves sleep quality. Dietary supplements are widely available, so it is debated which ones have a scientifically established beneficial effect on sleep quality. Many studies suggest the beneficial effects of melatonin and vitamin D on sleep quality [25].

Assessing sleep quality is another challenge in research in this area. Over the years, there has been a need for an appropriate tool to evaluate sleep quality, resulting in the development of the Pittsburgh Sleep Quality Questionnaire (PSQI). Studies that tested this tool showed that it is easy for patients to use, has adequate validation, and addresses the most critical elements affecting sleep quality (subjective sleep quality, sleep latency, sleep duration, habitual sleep efficiency, sleep disturbances, use of sleeping medication, and daytime dysfunction), which is reflected in the specific values obtained as scores assessing the subject’s sleep quality [26].

Education about sleep hygiene is also an essential aspect of research on sleep quality. However, it turns out that research results have been inconclusive, and individual knowledge about sleep hygiene does not always translate into higher sleep quality [6,27]. Many interventions, including, for example, short educational programs, did not have any beneficial effect on sleep quality [28,29,30]. The results show that the strength of evidence is low, while students are a group that consistently has problems with sleep quality [31]. However, there are also several papers available showing that possessed knowledge can have a beneficial effect on the quality of sleep [32,33]. This has been proven especially with longer educational programs aimed at sleep hygiene education [34,35,36].

The presented study is the result of discussions on the impact of possessed knowledge of sleep hygiene and diet on the quality of sleep. It indicates the respondents’ level of knowledge (taking into account their educational level). In addition to previous studies, an essential aspect of the presented research is the inclusion of the prevalence of supplementation, with an indication of the most commonly used supplements. The novelty of the study is that it was conducted at the end of the COVID-19 pandemic, during which significant adverse changes in sleep quality were observed [37], thus allowing analysis of recent trends in sleep quality which were undoubtedly impacted by the COVID-19 pandemic.

## 2. Materials and Methods

### 2.1. Subjects

The study was conducted in May 2021 through an online survey. Participation in the study was anonymous and voluntary. Inclusion criteria included: (1) informed consent, (2) age between 18 and 40, (3) working as a daytime laborer. Exclusion criteria included: (1) lack of informed consent, (2) age under 18 or over 40, (3) shift work, (4) a diagnosed medical condition that directly impacts sleep, e.g., obstructive sleep apnea, (5) narcotics addiction. Medical conditions or narcotics abuse could potentially impact sleep quality and may have interfered with the results, which is why only generally healthy participants were included in the study. A total of 402 subjects participated in the study, of whom 353 subjects were finally included in the analyses.

### 2.2. Procedure

Three self-reported measures were applied in the study. The first measured sleep quality, the second knowledge of sleep hygiene, and the last knowledge of supplementation to improve sleep quality. Moreover, respondents answered socio-demographic questions related to their current occupational and social status.

At the beginning of the survey, each respondent read the instructions and gave informed consent for participation and data processing. The study was approved by the local Bioethics Committee of the Medical University of Lublin (consent no. KE-0254/189/2021). The study was conducted in line with the directives of the Declaration of Helsinki on Ethical Principles for Medical Research. All participants gave informed consent agreement before the examination.

### 2.3. Measures

For sleep quality, the 6 components of the Pittsburgh Sleep Quality Questionnaire (PSQI) were used [26]. This questionnaire consists of 18 questions in which participants provide answers regarding subjective sleep quality, sleep latency (time needed to fall asleep), sleep duration, sleep disturbance, use of medication, and daytime sleepiness. The respondents’ answers are converted to raw scores according to a recognized answer key and then summed within each component. In addition, an overall sleep quality score is calculated which includes all components measured in the study. Each score obtained by the subjects is coded on a scale from 0 to 3, where 0—very good, 1—fairly good, 2—fairly bad, and 3—very bad. Thus, the overall score for the method ranges from 0 to 18 points, where a higher number of points indicates poorer sleep quality. According to the method’s standards, a score of more than 5 points means poor sleep quality, and more than 10 points indicates sleep disturbance. Studies on sleep quality conducted using the PSQI indicate that the tool is well validated and easily accessible to participants [38]. For the purpose of this study, a Polish version of the PSQI was used.

Additionally, the survey participants were asked about dietary supplements they had used for a minimum of one month to improve sleep quality. In Poland, medications are legally registered, clinically tested, and controlled in terms of doses and quality medical agents, while dietary supplements are agents that are commonly used and available; however, they do not require clinical tests or constant production control.

Knowledge about sleep hygiene was measured by a questionnaire which was developed by the research team based on the available literature. The questionnaire was originally distributed in the Polish language. Sleep hygiene knowledge was defined as knowledge covering basic issues that impact sleep quality such as going to bed at regular times, the length of naps during the day, avoiding distractions before bedtime, or proper preparation of the bedroom before bedtime, i.e., proper room temperature and humidity. The questionnaire consisted of 10 closed-ended questions which the respondents answered by indicating only one correct answer out of 5 available closed-ended options. Each respondent obtained 1 point for a correct answer and 0 points for an incorrect answer. The range of scores for this tool was from 0 to 10 points. The sample question was: “What is the optimal length of sleep for adults?” Respondents answered this example question by choosing one of five answers: (1) 5–7 h, (2) 7–9 h, (3) 8.5–9.5 h, (4) 9–10 h, (5) Don’t know. The full translated questionnaire is attached as Supplementary Material S1.

Knowledge about supplementation (including questions about supplements commonly used to improve sleep quality) and diet affecting sleep quality was measured with a method similar to the sleep hygiene questionnaire. Dietary knowledge refers to basic knowledge of the effects of diet (last meal before bed and its composition) and individual dietary components (alcohol, caffeine, beet juice, kiwi, milk, cherries) on sleep. Eight closed-ended, team-authored research questions based on the available literature were used, e.g., “Does eating cherries before bed affect sleep quality?” Respondents had 3 options to choose from: Yes/No/Don’t know. Respondents’ correct answers were coded as 1 point and summed to obtain an overall score. The range of scores was from 0 to 8 points. The questionnaire was originally in Polish. The full translated questionnaire is attached as Supplementary Material S2.

### 2.4. Data Analysis Strategy

Statistical data analysis was performed with SPSS software version 27. First, overall scores and descriptive statistics were calculated for the scales: PSQI, knowledge of sleep hygiene, and knowledge of diet and supplementation. At this stage, detailed scores for the 6 subscales of the PSQI were also calculated, and an analysis of the distribution normality was performed using the Kolmogorov–Smirnov test. A correlation analysis was then conducted using Pearson’s R coefficient between all variables in the study. The cut-off points used for the correlation coefficient were as follows: <0.40 as low, 0.40–0.69 as moderate, and ≥0.70 as high correlation. A *p*-value below 0.05 was considered significant. In the next step, a linear regression analysis for general sleep quality (PSQI) was conducted by including predictors such as knowledge of sleep hygiene, knowledge of diet and supplementation, use of medications, and use of supplements. The same relationships were tested for the overall PSQI by respondents’ education and place of residence. Moreover, additional detailed analyses were conducted for the particular component of sleep quality.

## 3. Results

### 3.1. General Characteristics of the Study Population

Among the 353 subjects analyzed in the study, 72.8% were women. The age of the subjects ranged from 18 to 40 years (mean 28.12 ± 6.08 years). Among all respondents, 41.6% had a master’s degree, 32.9% had a bachelor’s or engineering degree, and 25.5% had a high school degree or below. Respondents mainly resided in cities with up to 500,000 residents (41.3%) and cities with more than 500,000 residents (36%), while the fewest people lived in rural areas (22.7%).

### 3.2. Descriptive Statistics

Table 1 presents a detailed analysis of descriptive statistics and the normality distribution of all variables contained in the study. The mean score for general sleep quality was M = 6.03 (SD = 2.45). In the general PSQI, subjects obtained a score ranging from 0 to 14 points, with the highest possible score of 18 points. The mean scores for the sleep quality components oscillated around 1 point, except for the medication component (M = 0.26, SD = 0.68), which showed that subjects used medication infrequently. Scores on these scales ranged from 0 to 3 points, and analyses with the Kolmogorov–Smirnov test showed that, in all components, the distribution of scores was not close to normal (*p* < 0.05).

In the sleep hygiene knowledge test, participants achieved a score ranging from 1 to 9 points (M = 5.32, SD = 1.65), while, in the diet knowledge test, their results ranged between 0 and 8 points (M = 2.90, SD = 1.46). Respondents also answered additional questions about the use of medications and supplements to improve sleep quality. Supplements were used by 43.8%, while medications were used by 5.1% of respondents. Respondents indicated ashwagandha, magnesium, omega-3 acids, and melatonin as the most commonly used supplements.

### 3.3. Relationship between Knowledge and Use of Supplements and Sleep Quality

Respondents mostly declared that they did not take any dietary supplements (taken by 43.8%) or medications (taken by 5.1%). The agents that respondents took for a minimum of one month were: ashwagandha, omega-3, melatonin, CBD oil, 5-HTP, magnesium, GABA, l-theanine, multivitamin preparations, herbal preparations (lemon balm, lavender, chamomile), and hydroxyzine.

In the beginning, a correlation analysis between knowledge tests and sleep quality components was conducted (Table 2). The results revealed the presence of three statistically significant correlations, each of which was negative and low. Knowledge of sleep hygiene was significantly associated with sleep length (R = −0.17, *p* = 0.003) and overall sleep quality (R = −0.17, *p* = 0.005). In addition, dietary knowledge significantly correlated with time to fall asleep (R = −0.12, *p* = 0.026). Given the way the PSQI sleep quality score was calculated (see Section 2), any statistically significant correlation indicated that sleep quality improved as the level of knowledge increased.

### 3.4. Predictors of Overall and Individual Sleep Quality

For the next step, a linear regression analysis for the overall sleep quality and individual components of sleep quality was conducted (Table 3). Knowledge of sleep hygiene, knowledge of sleep-related diet, and use of supplements and medications were simultaneously entered as predictors.

For the general PSQI, the regression model had a good fit to the data, *F*(4,270) = 15.72, *p* = 0.001, and explained 18% of the variance. Detailed analyses revealed that knowledge of sleep hygiene was a significant negative predictor of sleep quality disorder (β = −0.13, *p* = 0.028), indicating that increased knowledge promotes better sleep quality. Statistically significant results were also obtained for the use of supplements (β = −0.20; *p* = 0.001) and the use of medications (β = −0.32, *p* = 0.001), which were also negative predictors of sleep quality, meaning that sleep quality increased with the use of medications or supplements.

**Figure 1:**
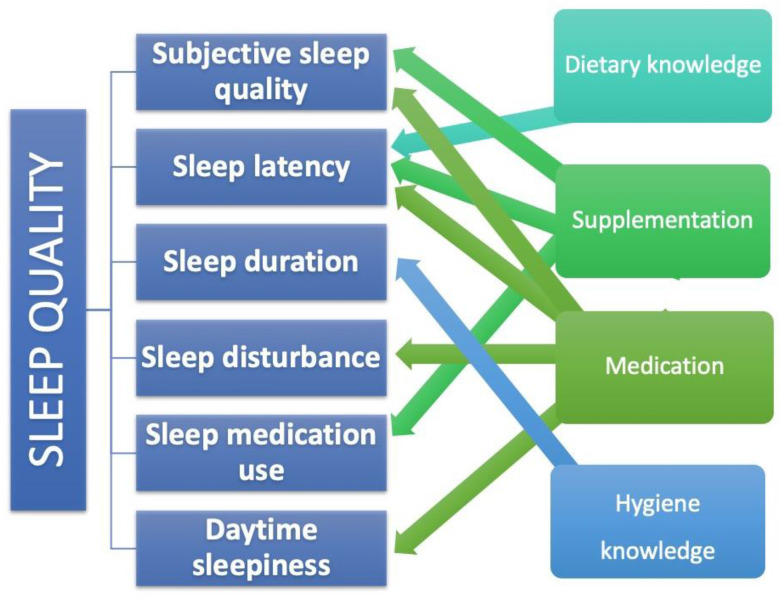
The impact of selected factors on the components of sleep quality.

Analysis of the detailed components of sleep quality showed that each model was a good fit for the data and explained between 2 and 10% of the variance. Knowledge of sleep hygiene was a significant predictor of sleep duration (β = −0.17, *p* = 0.007), and knowledge of diet was a significant predictor of sleep latency (β = −0.14, *p* = 0.026). In contrast, supplement use was a significant predictor of subjective sleep quality (β = −0.13, *p* = 0.024), sleep latency (β = −0.13, *p* = 0.029), and medication use (β = −0.33, *p* = 0.001). Another predictor tested in the model was medication use, which significantly predicted subjective sleep quality (β = −0.16, *p* = 0.006), sleep latency (β = −0.21, *p* = 0.001), sleep disturbance (β = −0.21, *p* = 0.001), and daytime dysfunction due to sleepiness (β = −0.12, *p* = 0.044). Each of the above relationships was negative, meaning that sleep quality improved as the level of the predictor increased. The simplified results are also presented in Figure 1.

### 3.5. Analysis by Educational Level and Place of Residence

For the general PSQI total score, linear regression analysis was also conducted by subgroups defined by educational level. The subjects were divided into three subgroups: those with high school education and below, those with a bachelor’s/engineering degree, and those with a master’s degree (Table 4). The analysis did not show any significant differences in the general sleep quality between the subgroups (*p* = 0.55). Model fit analysis showed that, in each subgroup, the regression model achieved a good fit to the data (*p* < 0.05) and explained between 13 and 21% of the variance. For those with a high school education, medications use was a significant predictor of sleep quality (β = −0.49; *p* = 0.001), while, among those with a bachelor’s/engineering degree, knowledge of sleep hygiene (β = −0.27; *p* = 0.016) and medication use (β = −0.49; *p* = 0.022) significantly predicted sleep quality. Among those with a master’s degree, supplements use (β = −0.32; *p* = 0.001) and medication use (β = −0.28; *p* = 0.002) were significantly associated with sleep quality. Negative associations between the predictors and the dependent variable meant that, as knowledge and substance use increased, the PSQI score decreased, i.e., the sleep quality of the subjects increased.

Predictors of sleep quality were also analyzed by place of residence. Respondents were divided into three subgroups: those living in rural areas, those in cities with up to 500,000 residents, and those in cities with over 500,000 residents. A model fit analysis showed that each of the tested models had a good fit to the data (Table 5) and explained between 16 and 29% of the general PSQI variance. Among rural residents, medication use was a significant predictor of sleep quality (β = −0.57; *p* = 0.001), and a similar effect was observed among participants from cities with up to 500,000 residents (β = −0.36; *p* = 0.001). However, among people living in cities with more than 500,000 residents, only supplementation significantly predicted general PSQI (β = −0.38; *p* = 0.001).

## 4. Discussion

The main purpose of this study was to analyze individual as well as environmental factors associated with sleep quality among young adults aged 18–40 years old. The study included dietary knowledge, sleep hygiene knowledge, and the use of supplements and medications as the main predictors of sleep quality. Moreover, additional analyses were conducted for environmental factors such as place of residence and the educational level of the participants. The COVID-19 pandemic impacted sleep routines and quality [39]. These changes have even been described as COVID-19 insomnia [40]. It should also be noted that the COVID-19 pandemic had an impact on mental health symptoms, including fatigue and sleep disorders or even cognitive disorders (memory disorders, concentration problems, or brain fog). It is estimated that, during the pandemic, the prevalence of depression and anxiety ranged from 20 to 35% of the general population. Furthermore, anxiety and depressive symptoms could persist for longer, even several months after COVID-19 infection, thus affecting sleep quality [41]. This sort of disturbance may have led to increased demand for methods of improving sleep quality. There have been studies that indicated the role of physical activity in this context [42]. However, this is the first study that analyzed the role of the above-mentioned factors (supplementation and knowledge) in affecting sleep quality at the end of the COVID-19 pandemic.

First, an overall assessment of the participants’ sleep quality was made through the overall score of the PSQI questionnaire, the mean score of which (M = 6.03, SD = 2.45) indicated the poor sleep quality of the participants. This confirms the result of the 2020 systematic review, which indicated that people over the age of 18 years old have problems with sleep quality [43]. It should also be noted that the study was conducted during the COVID-19 pandemic, and some studies indicated that the pandemic contributed to poor sleep quality. For example, in a study by Lipert et al., as many as 64% of the participants had poor sleep quality [37].

The analysis of predictors of overall sleep quality showed the statistically significant effect of sleep hygiene knowledge, use of medications, and use of dietary supplements. All of these variables indicated a negative relationship, meaning that, as their levels increased, overall sleep quality improved. This result differs from those of some scientific papers examining these relationships [28,44]. The discrepancies might be caused by the fact that the study by Mazar et al. was based only on a population of 87 medical students, while the study by Peach et al. was based on 218 college students; thus, the aspects of theoretical knowledge and educational level and sleeping quality might have been impacted by this fact [28,44]. This study enrolled a more heterogeneous and larger group of young adults which could better reflect the general young adult population; however, it is worth noting that most of the participants of the presented study were women. What is more, the results of the mentioned studies are in line with the subgroup analysis regarding the educational level in the presented study, where sleep hygiene knowledge turned out to be a predictor of sleep quality only in bachelor’s degree graduates. Moreover, there are available examples in the literature showing that knowledge has a beneficial effect on improving sleep quality and, similarly, that supplementation can also improve sleep quality [25,31].

A study conducted by researchers from the University of South Australia showed that personal motivation is a very important factor impacting the adoption of sleep hygiene measures regardless of knowledge, which shows how important it is to properly plan a sleep hygiene education program [45].

In the presented study, the model was tested in subsequent analyses with the same predictors but for individual components of sleep quality, which consisted of sleep length, sleep disturbance, sleep latency, daytime dysfunction, sleep efficiency, and subjective sleep quality [26]. The results revealed that greater knowledge of sleep hygiene promotes longer sleep duration. Scientific studies examining the relationship between knowledge and sleep duration indicated an improvement in sleep duration especially on weekend days which are free from work and study [46,47].

In contrast, better dietary knowledge improves the aspect of sleep latency. Despite the many pieces of research analyzing the effects of specific food components on sleep, there is a lack of information on the effect of possessed dietary knowledge on sleep quality. These results suggest that those with such knowledge put it into practice (e.g., taking dinner with a high glycemic index or using foods rich in tryptophan), which leads to reduced sleep latency [48,49].

The use of supplements was positively associated with an overall subjective assessment of sleep quality and the use of medications with less daytime sleepiness. Both drug and supplement uses were associated with shorter sleep duration. These results confirm the effects of supplements and drugs on sleep documented in the literature [25,50]. However, it should be noted that the sleep medications used, despite their beneficial effects on sleep, may carry some side effects, such as a lower physical and mental quality of life (QOL) [51]. Moreover, due to the cross-sectional design of this study, it cannot be verified whether the supplements and medications use is the result of poor sleep quality or whether their overuse impacts sleep quality. Supplements were used by 43.8% of participants, while medications were used by 5.1% of respondents, which shows that dietary supplement use is very popular in this group. The most common supplements used by the respondents were ashwagandha, magnesium, omega-3 acids, and melatonin. There is no clear evidence regarding the beneficial effect of magnesium supplementation on sleep quality [52]. Such an effect is mainly observed in people with magnesium deficiencies. A study by Nielsen et al. showed that magnesium deficiencies can lead to poorer sleep quality [53]. On the other hand, a balanced diet based on vegetables, fruits, whole grains, and low-fat dairy products is the best solution in the first place. Omega-3 fatty acids are involved in the conversion of serotonin to melatonin, which can improve sleep quality [54]. According to current knowledge, no specific role can be attributed to ashwagandha supplementation in improving sleep quality [25]. Melatonin is the main regulator of the diurnal cycle, so its supplementation may be warranted, especially for shift work or airplane flights to destinations with different time zones (to avoid jet lag) [17,55]. It should also be noted that cranberries, pistachios, walnuts, red rice, strawberries, or cherries are good food sources of melatonin [56].

The analysis of the impact of the above-mentioned environmental factors (place of residence and educational level) on sleep quality showed that the use of medications, regardless of educational level, was associated with better sleep quality, while the use of supplements promoted sleep quality only in those with higher education. Knowledge of sleep hygiene was only significant among those with a bachelor’s/engineering degree. Many publications have shown a correlation between lower levels of education and sleep quality, again suggesting an important role for sleep hygiene education [57,58,59]. The place of residence was also significant for sleep quality. In the present study, the use of medications was found to benefit sleep quality for residents of rural areas and cities with up to 500,000 residents, while the use of supplements was found to benefit residents of cities with more than 500,000 residents. Other predictors in these subgroups were found to be statistically insignificant. Socioeconomic status is a significant determinant of sleep quality, as one’s education, wealth level, and whether one lives alone or with a family have an impact on sleep quality [60,61,62]. Nonetheless, further publications analyzing the impact of place of residence on sleep quality are needed.

### Limitations of the Study

The presented results indicate further directions for research; however, this study has its limitations. It is a questionnaire-based study, so the results depended on the correctness of completion and the sincere responses of the subjects. On the other hand, no other tools are available to assess overall sleep quality for epidemiological purposes. Moreover, there are no available validated tools for sleep hygiene and dietary knowledge assessment, so, as well as the PSQI, the study was also based on ad hoc authored questionnaires. This is also a cross-sectional study, so the observed correlations are correlational in nature and do not allow me to make clear conclusions about cause-and-effect relationships. Further prospective, and ideally interventional, studies will allow for clear verification of the results obtained. The study considered a limited, small number of predictors. The reason was to simplify the questionnaire as much as possible to improve respondents’ cooperation. A questionnaire that is too long could affect compliance. Another aspect is that most of the respondents were women, who were more keen on taking part in the survey, so the results should be interpreted with great caution. Further research focused on other predictors will allow further development in this area.

## 5. Conclusions

The study showed that it is important to conduct educational programs on sleep hygiene knowledge and sleep dietary knowledge. The results indicated a significant effect of possessed sleep hygiene knowledge on sleep length and overall sleep quality. Sleep dietary knowledge is also an important factor. This study showed that sleep dietary knowledge had an impact on falling asleep. In addition, attention should be paid to supplementation, which does not always have scientifically documented effects, but is still popular among young adults. It is important to remember that a well-balanced and varied diet should be the first choice before using supplementation. As the COVID-19 pandemic may have impacted not only short-term but also long-term sleep quality aspects, this topic should still be investigated in the future.

## Figures and Tables

**Table 1:** Descriptive statistics of all variables in the study.

Variable	M	SD	S	K	X_min_	X_max_	K-S *
General sleep quality	6.03	2.45	0.49	0.18	0	14	0.001
Hygiene knowledge	5.32	1.65	−0.17	−0.36	1	9	0.001
Diet knowledge	2.90	1.46	0.34	−0.14	0	8	0.001
Subjective sleep quality	1.16	0.59	0.27	0.45	0	3	0.001
Sleep latency	1.27	0.86	0.36	−0.44	0	3	0.001
Sleep duration	1.02	0.85	0.31	−0.82	0	3	0.001
Sleep disturbance	1.03	0.40	0.25	3.24	0	3	0.001
Sleep medication use	0.26	0.68	2.89	7.83	0	3	0.001
Daytime sleepiness	1.34	0.83	0.19	−0.48	0	3	0.001

Note. M—mean, SD—standard deviation, S—skewness, K—kurtosis, K-S—Kolmogorov–Smirnov test, *—*p*-value for test reported.

**Table 2:** Correlations between hygiene and dietary knowledge with sleep quality components.

	Subjective Sleep Quality	Sleep Latency	Sleep Duration	Sleep Disturbance	Sleep Medication Use	Daytime Sleepiness	General Sleep Quality
Hygiene knowledge	−0.09	−0.10	−0.17 **	−0.11	0.05	−0.11	−0.17 **
Diet knowledge	−0.03	−0.12 *	−0.10	−0.02	−0.03	−0.07	−0.10

Note. * *p* < 0.05, ** *p* < 0.01.

**Table 3:** Predictors of individual components of sleep quality.

Predictor	Model Fit				Regression Coefficients		
	*df*	*F*	*p*	*R^2^*	*β*	*t*	*p*
General sleep quality (PSQI)							
Hygiene knowledge	4270	15.72	0.001	0.18	−0.13	−2.21	0.028
Diet knowledge					−0.06	−0.92	0.357
Supplementation use					−0.20	−3.47	0.001
Medications use					−0.32	−5.76	0.001
Subjective sleep quality							
Hygiene knowledge	4296	4.43	0.002	0.04	−0.09	−1.39	0.167
Diet knowledge					−0.01	−0.17	0.868
Supplementation use					−0.13	−2.27	0.024
Medications use					−0.16	−2.77	0.006
Sleep latency							
Hygiene knowledge	4296	7.32	0.001	0.08	−0.04	−0.67	0.505
Diet knowledge					−0.14	−2.23	0.026
Supplementation use					−0.13	−2.20	0.029
Medications use					−0.21	−3.68	0.001
Sleep duration							
Hygiene knowledge	4296	2.71	0.030	0.02	−0.17	−2.70	0.007
Diet knowledge					−0.01	−0.11	0.912
Supplementation use					−0.05	−0.92	0.356
Medications use					−0.05	−0.90	0.367
Sleep disturbance							
Hygiene knowledge	4270	4.21	0.003	0.05	−0.11	−1.72	0.087
Diet knowledge					0.04	0.65	0.517
Supplementation use					−0.01	−0.14	0.892
Medications use					−0.21	−3.52	0.001
Sleep medication use *							
Hygiene knowledge	3296	11.89	0.001	0.10	0.06	0.96	0.338
Diet knowledge					−0.07	−1.18	0.238
Supplementation use					−0.33	−5.90	0.001
Daytime sleepiness							
Hygiene knowledge	4296	2.53	0.041	0.02	−0.09	−1.39	0.165
Diet knowledge					−0.05	−0.78	0.436
Supplementation use					−0.06	−0.99	0.323
Medications use					−0.12	−2.03	0.044

Note. * for the sleep medication use component of the PSQI, regular medication use was excluded from the analysis.

**Table 4:** Predictors of sleep quality according to education level.

Predictors	Model Fit				Regression Coefficients		
	*df*	*F*	*p*	*R* ^2^	*β*	*t*	*p*
High school education (*n* = 90)							
Hygiene knowledge	4.68	4.72	0.002	0.18	−0.03	−0.26	0.793
Diet knowledge					0.09	0.66	0.510
Supplementation use					0.01	0.08	0.933
Medications use					−0.49	−4.24	0.001
Bachelor’s degree (*n* = 116)							
Hygiene knowledge	4.87	4.27	0.003	0.13	−0.27	−2.46	0.016
Diet knowledge					−0.02	−0.15	0.881
Supplementation use					−0.20	−1.98	0.051
Medications use					−0.24	−2.33	0.022
Master’s degree (*n* = 147)							
Hygiene knowledge	4.11	8.52	0.001	0.21	−0.11	−1.23	0.222
Diet knowledge					−0.14	−1.54	0.127
Supplementation use					−0.32	−3.65	0.001
Medications use					−0.28	−3.24	0.002

Note. The use of medications and supplementation was coded: 0—does not use, 1—uses.

**Table 5:** Predictors of sleep quality according to place of residence.

Predictors	Model Fit				Regression Coefficients		
	*df*	*F*	*p*	*R* ^2^	*β*	*t*	*p*
Rural area (*n* = 80)							
Hygiene knowledge	4.58	6.85	0.001	0.29	0.02	0.16	0.873
Diet knowledge					−0.16	−1.24	0.219
Supplementation use					−0.02	−0.15	0.878
Medications use					−0.57	−4.93	0.001
City with up to 500,000 residents (*n* = 146)							
Hygiene knowledge	4.12	6.49	0.001	0.16	−0.12	−1.26	0.211
Diet knowledge					−0.03	−0.27	0.789
Supplementation use					−0.15	−1.73	0.087
Medications use					−0.36	−4.12	0.001
City with over 500,000 residents (*n* = 127)							
Hygiene knowledge	4.90	5.86	0.001	0.18	−0.13	−1.31	0.192
Diet knowledge					−0.08	−0.80	0.425
Supplementation use					−0.38	−3.82	0.001
Medications use					−0.16	−1.66	0.101

Note. The use of medications and supplementation was coded: 0—does not use, 1—uses.

## Data Availability

The data supporting this study’s findings are available from the corresponding author upon reasonable request.

## Supplementary Materials

The following supporting information can be downloaded at: https://www.mdpi.com/article/10.3390/nu15153354/s1, S1: The questionnaire for sleep hygiene knowledge and S2: The questionnaire for dietary and supplementation knowledge (S2).
