# Supplementary material for: The Relationship between Knowledge, Dietary Supplementation, and Sleep Quality in Young Adults after the COVID-19 Pandemic
Source: Nutrients. 2023 Jul 28;15(15):3354. doi: 10.3390/nu15153354 (PMC10421253; doi:10.3390/nu15153354)
Supplement: Supplementary file 1 [file nutrients-15-03354-s001.zip › nutrients-2511379-supplementary/nutrients-2511379 Supplementary Material S1.pdf]

**Author's questionnaire concerning sleep hygiene knowledge**

In this part of the survey, you will be asked to answer questions about determinants of sleep quality. Please do not check your answers or "score". The survey is anonymous, so you will not suffer any consequences for giving wrong answers. Try to answer according to your state of knowledge. If you guess an answer but are unsure, please mark the answer as "I don't know".

1. What is the optimal sleep length for adults?
   a) 5-7 h
   b) 7-9h
   c) 8,5 - 9,5h
   d) 9-10h
   e) I don't know

2. Does going to bed at regular times affect the quality of sleep?
   a) Yes, you should go to bed every day and wake up at consistent times
   b) Yes, one should go to bed before 24
   c) No, the total amount of sleep counts
   d) I don't know

3. How long at most should a daytime nap last?
a) Up to 15 minutes
b) Up to 20 minutes
c) Up to 30 minutes
d) Up to the hour
e) I don't know

4. Does blue light affect the quality of sleep?
a) Yes, it affects dopamine secretion
b) Yes, it affects melatonin secretion
c) Does not affect
d) I don't know

5. Which things emit blue light?
a) Computer and mobile phones
b) Sun
c) Energy-saving light bulbs
d) All answers
e) I don't know

6. What is the optimal temperature in the room where you sleep?
a) Under 20 °C
b) 20 - 26°C
c) 27 - 28°C
d) I don't know

7. What is the optimal humidity in the room where you sleep?
a) About 30%
b) About 40%
c) About 50%
d) I don't know

8. Is using the snooze option before getting out of bed good for our sleep?
a) Yes, it makes waking up easier
b) No, because it reduces the quality of sleep
c) It does not matter because it does not affect the quality of sleep
d) I don't know

9. Does smoking affect sleep quality?
a) In active smokers only
b) In active and passive smokers
c) In passive smokers only
d) I don't know

10. What would you do if you had to sleep off a sleepless night during the day?
a) I'll cover the windows, put in earplugs, inform the household members not to wake me up
b) I'll put on earplugs, take sleeping medicine, inform the household members not to wake me up
c) I'll cover the windows, put in earplugs, take a sleep aid supplement
d) I will do an intensive workout to feel more tired, I will put in earplugs
e) I don't know
