# Supplementary material for: The Relationship between Knowledge, Dietary Supplementation, and Sleep Quality in Young Adults after the COVID-19 Pandemic
Source: Nutrients. 2023 Jul 28;15(15):3354. doi: 10.3390/nu15153354 (PMC10421253; doi:10.3390/nu15153354)
Supplement: Supplementary file 1 [file nutrients-15-03354-s001.zip › nutrients-2511379-supplementary/nutrients-2511379 Supplementary Material S2.pdf]

Author's questionnaire concerning dietary components, supplementation and pharmacotherapy

The last part of the survey is about your knowledge of the effect of nutrition on sleep, and the use of sleep supplementation/pharmacotherapy. When answering these questions, please answer according to your state of knowledge. If you are unsure, do not guess. Consider supplements you have been using regularly for a minimum of one month.

1. How many hours before bedtime should the last meal be consumed?
   a) Less than one hour
   b) 1-3 hours
   c) More than 3 hours
   d) I don't know

2. Does alcohol improve sleep quality?
   a) Yes, because it helps you fall asleep
   b) No, because it disturbs shallow sleep
   c) No, because it disturbs deep sleep
   d) I don't know

3. Does beetroot juice improve sleep quality?
   a) Yes, because it reduces physiological fatigue
   b) Yes, because it helps you fall asleep
   c) Does not affect the quality of sleep
   d) I don't know

4. How much kiwi should you eat before bed to improve its quality?
   a) 1 kiwi 2 hours before going to bed
   b) 2 kiwis an hour before going to bed
   c) kiwis a day
   d) I don't know

5. Does drinking 1 cup of warm milk before bed help you fall asleep?
   a) Yes, because it has the right temperature
   b) Yes, because it is enough
   c) No, because it's too small
   d) I don't know

6. Does eating cherries before bed affect sleep quality?
   a) Yes
   b) No
   c) I don't know

7. How many hours before bedtime can you drink coffee so it does not disturb sleep quality?
   a) 3 - 4h
   b) 6 - 8h
   c) It does not matter
   d) I don't know

8. Which meal would you choose as the last meal before bedtime?
   a) Pasta salad with chicken in sweet and sour sauce
   b) Toast with avocado, egg and bacon
   c) Sandwiches with lean ham and spicy ketchup
   d) Salad with egg and tuna
   e)I don't know

9. Tick the supplements that affect the quality of sleep that you use (minimum one month):

- I don't use supplements
- Chamomile
- Ashwagandha
- L - ornithine
- Omega - 3
- Magnesium
- Lavender
- GABA

- CBD oil
- L - theanine
- melatonin
- 5 - HPP
- Baikal skullcap (skullcap)
- Multicomponent preparations
  Others:

10. Do you take prescription sleeping pills (e.g. Stilnox, Imovane, Zolpic, Nasen, Morfeo, Dobroson, and others)?
    a) Yes
    b) No

11. How often and for how long do you use a sleeping pill? (Question addressed to people who take these drugs, other people, please go to the next question).
    a) Every day for more than 4 weeks
    b) Daily, less than 4 weeks
    c) Only if necessary, ad hoc, longer than 4 weeks
    d) Only if necessary, ad hoc, shorter than 4 weeks

12. Have you started using supplements/sedatives during the pandemic?
    a) Yes
    b) No
13. Do you take other psychotropic drugs?
    a) Yes
    b) No
